# Real life real hope

**DOI:** 10.1186/2050-2974-1-S1-O12

**Published:** 2013-11-14

**Authors:** Helen Shepherd, Hayley Bennett, Simone Monty

**Affiliations:** 1 The Body Image and Eating Disorders Treatment and Recovery Service (BETRS), Australia

## 

Often it is mentioned by participants that having peers speak out about their personal journey has instilled a sense of hope and been an inspiring experience. From this evolved the Mentor program. The Body Image and Eating Disorders Treatment and Recovery Service (BETRS) Mentor Program has been developed to complement the professional expertise within the team. Our Mentors share their lived experience of an eating disorder with current patients of BETRS. The invaluable contribution to the service by our mentors has been the following:

- Supporting hopefulness and the recovery process by co-facilitating group sessions that focus on how they dealt with issues that are of particular concern to current patients.

- Strengthen relapse prevention by giving firsthand accounts of occasions when lapses have not signalled a relapse but have been incorporated into the recovery process.

- Mentors have been fostering motivation by encouraging behaviours that supported their own recovery and by assisting with the organisation and general care of the client group.

- By sharing their experience of having an eating disorder and providing evidence of hope during the course of recovery mentors have given family and carers and better understanding.

This abstract was presented in the **Care in Inpatient and Community Settings** stream of the 2013 ANZAED Conference.

